# Evaluation of the psychometric properties of the family adaptability and cohesion scale (FACES III) through item response theory models in students from Chile and Colombia

**DOI:** 10.1186/s40359-024-01526-x

**Published:** 2024-01-12

**Authors:** Lindsey W. Vilca, Víctor Díaz-Narváez, Wilson Pastén Hidalgo, Nicolás Van Niekerk Bakit, Paula Moreno Reyes, Yaneth Herazo-Beltrán, Aura Gauna-Quiñonez, Alejandro Reyes-Reyes, Luz Marina Alonso Palacio, Marco Cervantes Mendoza

**Affiliations:** 1 South American Center for Education and Research in Public Health, Universidad Norbert Wiener, Lima, Peru; 2 Faculty of Dentistry, Research Department, Universidad Andres Bello, Santiago, Chile; 3 Faculty of Health Sciences, Department of Kinesiology, Universidad de Atacama, Copiapó, Chile; 4 Department of Physiotherapy, Universidad Simón Bolívar, Barranquilla, Colombia; 5 Faculty of Social Sciences and Communications, School of Psychology, Universidad Santo Tomás, Concepción, Chile; 6 División de Ciencias de la Salud, Universidad del Norte, Barranquilla, Colombia; 7 División Humanidades y Ciencias Sociales, Departamento de Psicología, Universidad del Norte, Barranquilla, Colombia

**Keywords:** Family functioning, Cohesion, Adaptability, FACES III, Differential Item Functioning, Item response theory

## Abstract

**Background:**

A psychometric study of the Family Adaptability and Cohesion Scale (FACES III) has been conducted in Spanish-speaking countries from the perspective of the classical test theory. However, this approach has limitations that affect the psychometric understanding of this scale.

**Objective:**

Accordingly, this study used the item response theory to investigate the psychometric performance of the items. Furthermore, it evaluated the differential performance of the items for Colombia and Chile.

**Method:**

For this purpose, 518 health science students from both countries participated. Confirmatory Factor Analysis was used.

**Results:**

The study results revealed that the cohesion and adaptability items presented adequate discrimination and difficulty indices. In addition, items 5, 8, 13, 17, and 19 of cohesion indicated differential functioning between students from both countries, with Chilean students exhibiting a greater discriminatory power. Further, the Colombian group exhibited a greater discriminatory power for item 18 of adaptability.

**Conclusions:**

The study concluded that the items of FACES III indicated adequate psychometric performance in terms of their discriminative capacity and difficulty in Chile and Colombia.

## Introduction

Family functioning is a widely studied construct, where its importance for developing and maintaining mental health indicators in individuals has been demonstrated [1, 2]. Few studies have revealed that deficient family functioning is related to emotional problems such as anxiety and depression [3–5]. By contrast, more positive family functioning may favor better adjustment in youth [6–7] and lower psychological problems [8]. In this context, family functioning plays an essential role in the beginning of the university stage, since it favors better adaptation and coping with the demands of academic life [9]. In addition, a study conducted in China with medical students revealed that adequate family functioning is related to a lower presence of symptoms of depression and anxiety [10]. Similarly, another study conducted in the same country on medical, nursing, and medical technology students reported that good family functioning is associated with decreased risks of distress and stress [11]. Furthermore, another study conducted in the United States with nursing students revealed that better family functioning is related to lower stress, anxiety, and depression [12]. In Nigeria, a study conducted with health sciences students revealed that negative family functioning is associated with a higher level of depression [13]. In Latin America, a study conducted with Colombian medical students demonstrated that deficient family functioning is a risk factor for psychological distress [9]. Accordingly, another study conducted in the same country indicated that family functioning is a predictor of academic achievement [14]. A study conducted in Chile reported that family functioning is a protective factor against risk behaviors in students [15]. Therefore, adequately measuring family functioning in health science students is crucial. In relation to this, the Family Adaptability and Cohesion Scale (FACES) is most widely used to study this construct, of which different versions have been developed. Among them, FACES III is the most used, enabling a linear assessment of family functioning from the circumplex model [16].

Numerous studies conducted in Latin America have examined the psychometric performance of FACES III. In Argentina, a confirmatory factor analysis (CFA) was used to examine the factorial structure of the scale [17]. In Mexico, an exploratory factor analysis (EFA) was used to examine the psychometric performance of the scale [18]. In Chile, the EFA approach was used to examine the factorial structure of the scale [19]. A second-order CFA was used in another study conducted in the same country [20]. In Peru, a combination of EFA and CFA was used to examine scale performance [21]. Similarly, another study used EFA and CFA to examine the cohesion and adaptability dimensions of FACES III [22]. However, the evidence has not presented psychometric studies in the countries mentioned, extrapolating to Latin America that analyzes the internal structure of FACES III.

As indicated in previous studies, all approaches have been based on the classical test theory (CTT), such as EFA and CFA. However, the CTT has severe limitations [23]: (a) lack of invariance of the results with respect to the instrument used and (b) lack of invariance of the psychometric properties of the tests with respect to the group used to calculate them. Therefore, given the above findings, it can be explained why the factorial structure of FACES III is not the same in the different studies that have analyzed its structure. Accordingly, item response theory (IRT) presents three fundamental advantages [24]: (a) Invariance of the item parameters, i.e., the item parameters do not vary, even if the respondents differ; (b) invariance of the trait parameter of the respondent concerning the instrument used to calculate it, i.e., the ability level of the respondent does not depend on the test; and (c) provision of local measures of accuracy through the item information curve (IIC) and the test information curve (TIC). These features provide a detailed knowledge of the area in which the trait measured by the test is best being measured. In other words, it enables us to know for which level of the trait the instrument is best designed. In addition, it enables us to examine the differential analysis of the items between groups. This ensures more reliable comparisons to be made between those evaluated.

For all these reasons, the general objective of this research is to study the psychometric functioning of FACES III using Item Response Theory (IRT). Specifically, (a) the degree of discrimination and difficulty of the FACES III items will be studied, and (b) the differential functioning of the items between students from Colombia and Chile will be evaluated.

## Method

### Design

The present study used an instrumental design since the psychometric performance of a measurement instrument was analyzed [25].

### Participants

The study involved 518 physiotherapy and kinesiology students from universities in Colombia (Universidad Simón Bolívar) and Chile (Universidad de Atacama). Table 1 presents that the average age of participants living in Colombia is 20.1 (*SD* = 3.4) and that of participants in Chile is 21.8 (*SD* = 4.0). As indicated in the table, both countries comprise a higher proportion of women (Colombia = 84.1%; Chile = 53.4%) than that of men (Colombia = 15.9%; Chile = 46.6%). In addition, 63.1% are physiotherapy major, and 36.9% belong to the kinesiology major. Finally, both countries constitute students from different academic years.

**Table 1:** Demographic characteristics of the participants

Sociodemographic data	Colombia (*n* = 327)	Chile (*n* = 191)
Age (M ± SD)	20.1 ± 3.4	21.8 ± 4.0
Sex, *n* (%)		
Male	52 (15.9%)	89 (46.6%)
Female	275 (84.1%)	102 (53.4%)
Major, *n* (%)		
Physiotherapy	327 (63.1%)	‒
Kinesiology	‒	191 (36.9%)
Academic year		
First year	95 (29.1%)	51 (26.7%)
Second year	107 (32.7%)	38 (19.9%)
Third year	71 (21.7%)	40 (20.9%)
Fourth year	54 (16.5%)	51 (26.7%)
Fifth year	‒	11 (5.8%)

### Instrument

#### Family cohesion and adaptability evaluation scale (FACES III)

The study used the version adapted to Spanish in Chile [19], comprising 20 items that measure two dimensions: cohesion (1, 4, 5, 8, 10, 11, 13, 15, 17, and 19)and adaptability (2, 3, 6, 7, 9, 12, 14, 16, 18, 20). The items present five response categories scored as follows: never (0), almost never (1), rarely (2), frequently (3), and almost always (4). The scale does not present inverse items; therefore, a higher score in each dimension indicates a higher level of cohesion or adaptability, as the case may be.

### Procedure

For the study, approval was obtained from the ethics committee of the Universidad de San Sebastián, Chile (Resolution N° 2/2015 and N° 83/ 2020), and the standards established in the Helsinki declaration were followed [26]. The data were obtained in November 2019, and the collection process was the same for both countries.

For data collection, non-probabilistic convenience sampling was used. A virtual form was applied in classrooms. In the online form, informed consent was presented first. Followed by the study objectives and contact information for the study coordinators. The students acquired access to FACES III questions only after providing informed consent. During the data collection process, data confidentiality and the opportunity to withdraw from the evaluation at any time were ensured.

### Data analysis

First, compliance with the main assumptions of the IRT was evaluated. A separate graded response model (GRM) was fitted for each dimension of FACES III to meet the unidimensionality assumption. The G2 index [27], specifically Cramer’s V coefficient, which takes values between − 1 and 1, was used to evaluate the assumption of local independence of the items [28]. A large absolute value indicates a potential case of local dependence [29]. Compliance with the monotonicity assumption was also inspected using the raw residue plots [30].

A GRM [31], specifically an extension of the 2-parameter logistic model (2-PLM) for ordered polytomous items [32], was used to calculate the IRT models. The C2 test developed for ordinal items [33] was used to calculate the model fit. The following fit criteria were used: Root mean square error of approximation, RMSEA ≤ 0.06 [34] and Standardized root mean square residual, SRMSR ≤ 0.05 [35]. Comparative Fit Index (CFI) and Tucker-Lewis Index (TLI) values were also considered using the same fit criterion (≥ 0.95) employed in SEM models [36]. The generalized S-X2 index and its corresponding RMSEA were used as a measure of effect size to assess item fit [37].

In the GRM models, two types of parameters were calculated: discrimination (a) and difficulty (b). The discrimination parameter determines the slope at which item responses change as a function of the level in the latent trait. The item difficulty parameters determine how much of the latent trait the item requires to be answered. As the scale comprises five response categories, there are four difficulty calculations, one per threshold. The calculations for these four thresholds indicate the level of the latent variable at which an individual has a 50% chance of scoring at or above a particular response category. The following graphs representing item and test performance for each latent trait were also calculated: item characteristic curve (ICC), test characteristic curve (TCC), IIC, and TIC.

We used the likelihood ratio approach for ordinal items to assess differential item functioning (DIF) according to the participants’ country [29]. Under this approach, two models were calculated: (a) a no-DIF model, where all item parameters are invariant between groups, and (b) another DIF model, where item parameters can be unequal between groups. The no-DIF (reduced model) and DIF (full model) models were compared using the log-likelihood ratio test with the ANOVA function to test for possible differences in item parameters between groups. In this comparison, the null hypothesis establishes that there is no DIF, i.e., the parameters of the items are equal between countries. A *p* value < 0.05 was used to reject the null hypothesis.

The ‘mirt’ and ‘ltm’ packages [28, 38] were used to calculate the GRM models and DIF analysis. The RStudio environment [39] for R [40] was used in all cases.

## Results

### Descriptive analysis of the items

Table 2 indicates that in the cohesion dimension, item 13 (‘family members support each other in difficult times’) presents the highest average score (*M* = 3.58). That is, most participants agree with this statement. In the adaptability dimension, item 9 (‘family members are free to express themselves’) presents the highest average score (*M* = 3.46). That is, most participants agree with this statement. On the other hand, it is observed that the asymmetry and kurtosis of the items show a distribution moderately different from a normal distribution. (As < ± 2; Ku < ± 7) [41]. Furthermore, the response categories of all the items have been answered by the participants.

**Table 2:** Descriptive analysis of the items and response rate of the items

Items	M	SD	g1	g2	Response rate				
					0	1	2	3	4
1. Family members feel emotionally close to each other.	3.33	0.84	-1.21	1.12	0.6%	2.9%	12.2%	31.9%	52.5%
2. Children participate in problem solving.	2.74	1.10	− 0.68	− 0.14	4.6%	8.1%	24.7%	33.8%	28.8%
3. In our family, discipline (rules, obligations, consequences, punishments) is fair.	3.19	0.94	-1.20	1.21	1.7%	4.2%	13.1%	35.3%	45.6%
4. All family members participate in decision-making.	2.95	1.02	− 0.88	0.37	3.1%	5.2%	21%	35.3%	35.3%
5. Family members ask each other for help.	3.38	0.86	-1.48	1.98	1%	2.9%	10.4%	28.2%	57.5%
6. In the development of discipline guidelines (rules, obligations) we take into account the opinion of the children.	2.85	1.09	− 0.83	0.06	4.2%	7.5%	20.5%	34.4%	33.4%
7. When problems arise, we negotiate to find a solution.	3.01	1.03	-1.05	0.69	3.1%	6.2%	15.1%	38.4%	37.3%
8. In our family, we do activities together regularly.	2.91	1.09	− 0.76	− 0.24	2.9%	8.7%	21%	29.2%	38.2%
9. Family members are free to express themselves.	3.46	0.80	-1.83	4.02	1.4%	1.5%	6.9%	30.3%	59.8%
10. In our family we usually meet in the same place (kitchen, living room, or other space).	3.17	1.03	-1.19	0.86	2.7%	4.4%	16%	27%	49.8%
11. Family members like to spend our free time together.	2.77	1.09	− 0.59	− 0.42	3.1%	10.6%	23.4%	32%	30.9%
12. In our family, it is easy for all of us to express our opinion.	3.09	0.97	− 0.99	0.65	2.1%	3.9%	18.7%	33.6%	41.7%
13. Family members support each other in difficult times.	3.58	0.76	-2.24	5.61	1%	2.1%	4.6%	22.4%	69.9%
14. In our family, we try new ways to solve problems.	3.02	0.96	− 0.84	0.14	1.2%	7.1%	16.8%	38%	36.9%
15. Family members share interests and hobbies.	2.81	1.04	− 0.67	− 0.12	2.7%	8.7%	22.6%	36.7%	29.3%
16. We all have a say in family decisions.	2.97	1.04	− 0.88	0.24	2.9%	5.8%	20.7%	32.8%	37.8%
17. Family members consult each other about our personal decisions.	2.58	1.13	− 0.45	− 0.50	5.2%	11%	29.3%	29.5%	24.9%
18. Parents and children talk about punishments and rules.	2.60	1.21	− 0.62	− 0.42	8.3%	8.3%	25.9%	30.1%	27.4%
19. Family unity is a primary concern.	3.09	1.10	-1.16	0.54	3.7%	7.1%	13.3%	28.4%	47.5%
20. Family members discuss our problems and feel good about the decisions made together.	2.80	1.13	− 0.69	− 0.33	3.9%	10.4%	20.7%	31.9%	33.2%

### Item calibration with the GRM (2-PML)

The assumption of unidimensionality for the dimensions was empirically supported using a CFA: cohesion (χ^2^ = 172.43; df = 35; CFI = 0.97; TLI = 0.96; RMSEA = 0.087 [90% CI 0.074 ‒ 0.100]) and adaptability (χ^2^ = 138.99; df = 34; CFI = 0.97; TLI = 0.97; RMSEA = 0.077 [90% CI 0.064 ‒ 0.091]). Regarding the assumption of local independence, the standardized Cramer’s V values ranged from − 0.153 to 0.166 for the cohesion dimension. The Cramer’s V standardized values ranged from − 0.166 to 0.155 for the adaptability dimension. Therefore, the fulfillment of the assumption of local independence of the items for both the dimensions was confirmed. In addition, the raw residual plots for the items of both dimensions did not indicate a substantial deviation from monotonicity.

A GRM, specifically an extension of the 2-PLM for ordered polytomous items, was used to calculate the models. The GRM model for the Cohesion dimension presents acceptable fit indices (C2[df] = 13.70[5]; *p* <.05; RMSEA = 0.058[95% CI 0.022 ‒ 0.095]; SRMRS = 0.051; TLI = 0.90; CFI = 0.93). Similarly, the model for the Adaptability dimension presented adequate fit indices (C2[df] = 6.12[5]; *p* >.05; RMSEA = 0.020[95% CI 0.000 ‒ 0.067]; SRMRS = 0.054; TLI = 0.96; CFI = 0.99). Furthermore, Table 3 indicates that the items of both dimensions present *p*-values associated with the S-χ2 less than 0.001 and small RMSEA values (< 0.029). Accordingly, the items of both dimensions presented adequate adjustment indexes in the GRM model.

**Table 3:** Parameters of the items of the GRM models for the dimensions of FACES III

Dimension	Item	Item Parameter					Item fit indices		
		a	b_1_	b_2_	b_3_	b_4_	S_X2	RMSEA	*p*-value
Cohesion	1	1.58	-4.13	-2.85	-1.48	− 0.07	42.190	0.020	0.188
	19	1.55	-2.80	-1.88	-1.04	0.09	50.109	0.000	0.549
	5	1.67	-3.64	-2.63	-1.49	− 0.24	47.390	0.029	0.050
	17	1.57	-2.50	-1.41	− 0.14	0.97	60.220	0.027	0.052
	8	2.11	-2.54	-1.54	− 0.54	0.41	36.062	0.000	0.689
	13	2.06	-3.31	-2.57	-1.87	− 0.63	34.696	0.000	0.483
	4	1.31	-3.24	-2.28	− 0.83	0.61	52.549	0.000	0.492
	11	2.46	-2.40	-1.34	− 0.39	0.61	37.389	0.005	0.451
	10	1.66	-2.88	-2.09	-1.00	0.02	41.462	0.000	0.736
	15	2.23	-2.50	-1.50	− 0.51	0.68	40.485	0.005	0.449
Adaptability	7	2.18	-2.51	-1.71	− 0.84	0.43	44.79	0.013	0.316
	12	1.79	-2.98	-2.20	− 0.88	0.33	48.90	0.019	0.186
	2	1.18	-3.11	-2.04	− 0.54	0.99	61.30	0.016	0.231
	16	1.92	-2.66	-1.78	− 0.63	0.45	42.39	0.000	0.736
	6	2.01	-2.37	-1.56	− 0.54	0.59	52.42	0.012	0.343
	18	1.61	-2.06	-1.39	− 0.23	0.88	45.71	0.000	0.810
	14	1.89	-3.34	-1.89	− 0.85	0.48	36.90	0.000	0.799
	3	1.14	-4.09	-2.87	-1.52	0.24	69.50	0.023	0.089
	20	1.88	-2.50	-1.42	− 0.46	0.61	49.29	0.007	0.421
	9	1.74	-3.35	-2.76	-1.73	− 0.28	46.69	0.027	0.072

Note. a = discrimination parameters; b = difficulty parameters

Regarding the parameters of the GRM cohesion model, Table 3 indicates that all the discrimination parameters of the items are above the value of 1.35, generally considered a high level of discrimination [42]. Furthermore, Table 3 demonstrates that the adaptability items present adequate discrimination indexes (> 1.35), except for items 2 (1.18) and 3 (1.14) indicating moderate levels. Concerning the difficulty parameters, all threshold estimators increased monotonically. That is, a more significant presence of the latent trait is required to answer the higher response categories.

**Fig. 1:**
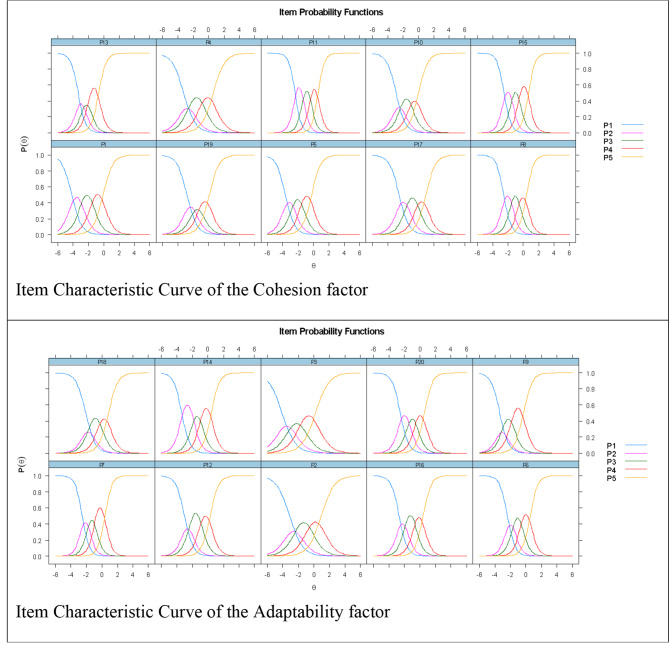
Item characteristic curve of FACES III

Figure 1 depicts that the response alternatives of the items are monotonically related to the levels of cohesion and adaptability, respectively. That is, as one moves from left to right in the ICCs, the probability of choosing a response category increase and then decreases as responses move to the next higher category.

**Fig. 2:**
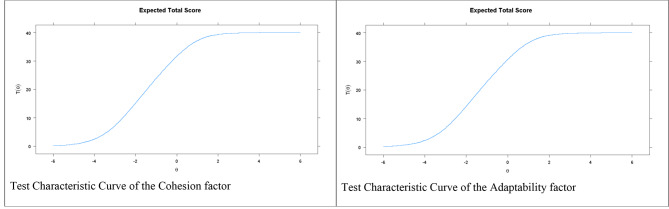
Test characteristic curve of FACES III

**Fig. 3:**
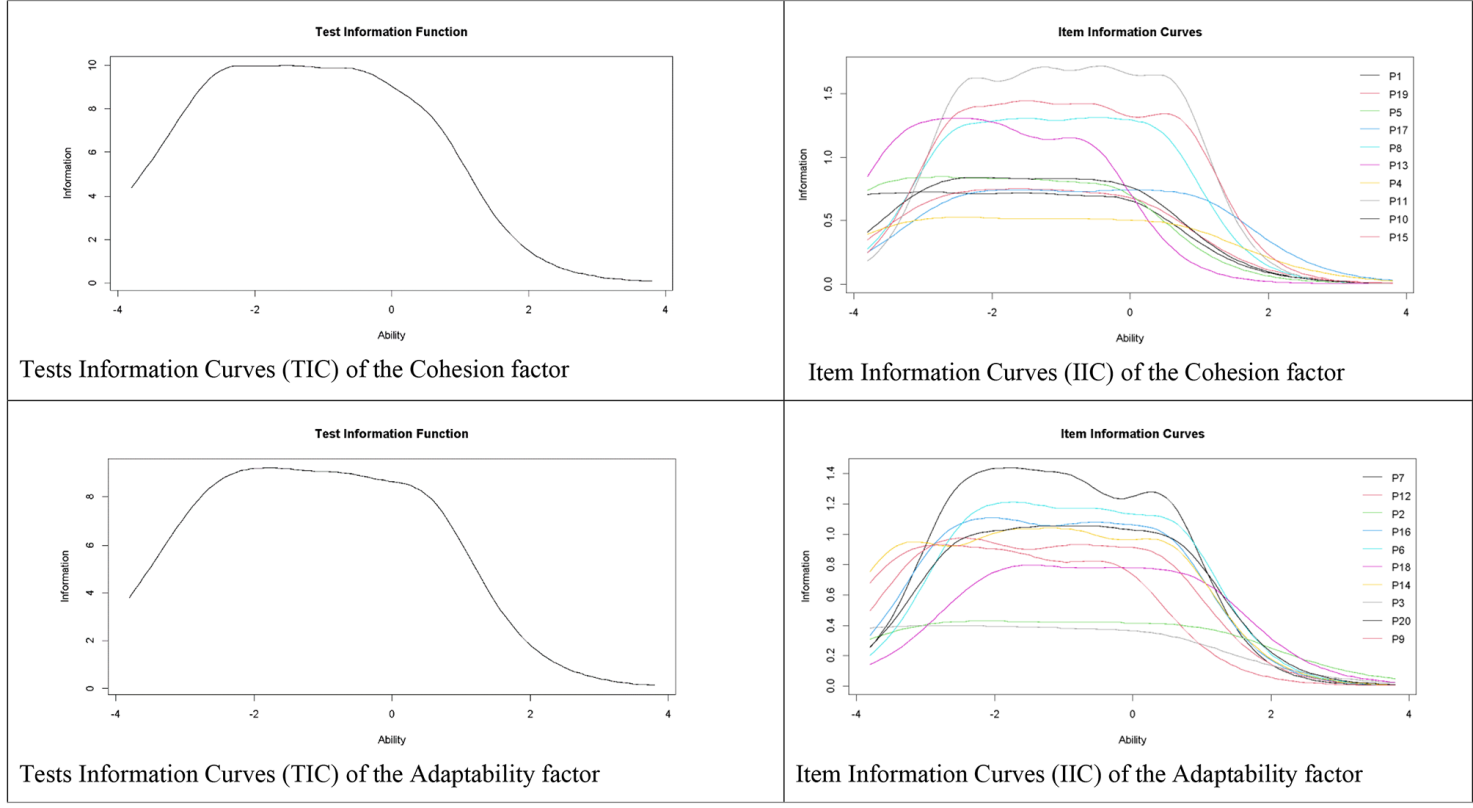
Item and test information curves for FACES III

Figure 2 demonstrates a sharp increase in the total scores of FACES III as the actual level of cohesion and adaptability increases. Figure 3 depicts the IIC and TIC. For cohesion in the IIC, items 6 and 7 are the most accurate items of the scale for assessing the latent trait. In addition, the TIC indicates that the test is more reliable (accurate) in the scale range between − 2.5 and 0.5. Regarding adaptability, the IIC indicates that items 11 and 19 are the most accurate items of the scale for assessing the latent trait. In addition, the TIC indicates that the test is more reliable (accurate) in the range of the scale between − 2.5 and 0.5.

### Differential item functioning (DIF)

**Fig. 4:**
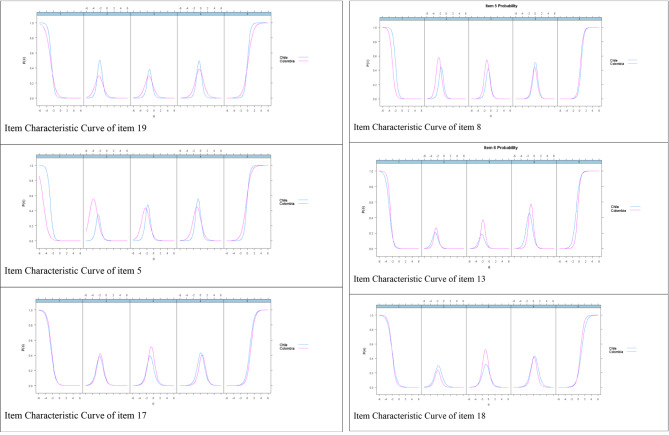
Item characteristic curve with DIF of FACES III

Table 4 depicts the differential analysis of the items between the Chilean and Colombian participants. For cohesion, items 19, 5, 17, 8, and 13 present a differential performance between both groups, with a greater discriminatory power for students from Chile. However, with regard to item 13, the Colombian group has a greater discriminatory power. For the other cohesion items, the ANOVA analysis indicates no presence of DIF (*p* > .05). With respect to adaptability, only item 18 shows a differential performance between the groups, with a greater discriminatory power for the Colombian group. These differences can also be observed in the ICCs of these items (see Fig. 4).

**Table 4:** Differential item functioning of the cohesion and adaptability factors

Ítem	Chile					Colombia					AIC	SABIC	HQ	BIC	χ^2^	df	*p*
	a	b_1_	b_2_	b_3_	b_4_	a	b_1_	b_2_	b_3_	b_4_							
1	1.70	-4.01	-2.81	-1.45	− 0.01	1.64	-3.82	-2.62	-1.36	− 0.09	11324.02	11385.34	11418.94	11566.27	1.48	5	0.914
19	2.30	-2.59	-1.65	− 0.95	− 0.01	1.35	-2.79	-1.89	− 0.99	0.19	11311.78	11373.10	11406.69	11554.03	13.73	5	0.017
5	2.28	-2.74	-2.12	-1.22	− 0.12	1.43	-4.72	-2.97	-1.67	− 0.32	11315.16	11376.48	11410.07	11557.41	10.90	5	0.046
17	1.61	-2.43	-1.43	− 0.40	0.75	1.74	-2.26	-1.24	0.05	1.04	11312.22	11373.54	11407.14	11554.47	13.28	5	0.021
8	2.31	-2.00	-1.19	− 0.42	0.54	2.21	-2.91	-1.71	− 0.60	0.26	11306.78	11368.10	11401.69	11549.03	18.73	5	0.002
13	1.77	-2.93	-2.45	-2.03	− 0.91	2.41	-2.62	-2.16	-1.51	− 0.43	11311.52	11372.84	11406.43	11553.77	13.99	5	0.016
4	1.44	-3.19	-2.17	− 0.85	0.62	1.36	-2.97	-2.13	− 0.73	0.55	11323.06	11384.38	11417.97	11565.31	2.45	5	0.783
11	2.36	-2.15	-1.18	− 0.34	0.75	2.85	-2.34	-1.33	− 0.41	0.43	11314.73	11376.05	11409.64	11556.98	10.78	5	0.056
10	1.81	-2.66	-2.20	-1.17	− 0.10	1.75	-2.72	-1.81	− 0.79	0.12	11315.88	11377.20	11410.8	11558.13	9.63	5	0.086
15	2.14	-2.53	-1.53	− 0.43	0.70	2.61	-2.22	-1.34	− 0.50	0.57	11322.32	11383.63	11417.23	11564.56	3.19	5	0.670
7	2.79	-2.41	-1.51	− 0.83	0.40	1.96	-2.62	-1.90	− 0.92	0.35	11912.04	11973.36	12006.95	12154.28	7.87	5	0.163
12	1.56	-3.09	-2.24	-1.03	0.37	2.08	-2.87	-2.16	− 0.84	0.19	11914.49	11975.81	12009.4	12156.74	5.42	5	0.366
2	1.03	-3.39	-2.39	− 0.67	1.22	1.36	-2.88	-1.83	− 0.53	0.73	11913.93	11975.25	12008.85	12156.18	5.98	5	0.308
16	1.77	-3.14	-2.03	− 0.82	0.26	2.12	-2.41	-1.64	− 0.55	0.47	11912.41	11973.73	12007.33	12154.66	7.50	5	0.186
6	2.28	-2.10	-1.38	− 0.57	0.42	1.88	-2.60	-1.74	− 0.59	0.62	11914.66	11975.98	12009.57	12156.90	5.26	5	0.385
18	1.44	-2.05	-1.19	− 0.29	1.00	1.83	-2.07	-1.55	− 0.28	0.67	11905.16	11966.48	12000.08	12147.41	14.75	5	0.011
14	1.93	-3.38	-1.90	− 0.76	0.52	1.94	-3.29	-1.91	− 0.97	0.33	11915.73	11977.05	12010.64	12157.98	4.18	5	0.523
3	1.29	-3.25	-2.33	-1.18	0.31	1.08	-4.93	-3.31	-1.80	0.10	11912.22	11973.53	12007.13	12154.47	7.70	5	0.174
20	2.16	-2.37	-1.53	− 0.62	0.47	1.79	-2.56	-1.37	− 0.40	0.62	11913.06	11974.38	12007.98	12155.31	6.85	5	0.232
9	1.85	-3.10	-2.70	-1.92	− 0.49	1.76	-3.42	-2.73	-1.62	− 0.20	11912.46	11973.78	12007.38	12154.71	7.45	5	0.189

## Discussion

This study evaluated the performance of FACES III items based on IRT. A CFA was performed for each dimension separately to comply with the assumption of one-dimensionality, indicating that each factor presents adequate fit indices to the data. This approach is similar to what has been done in other studies [43, 44], including for those studies on FACES III [22]. The local independence of the items and the fulfillment of the monotonicity assumption were also demonstrated, all of which guarantee the veracity of the calculations made [45].

Regarding the cohesion factor, all the items presented adequate discrimination indexes, showing that they allow us to adequately differentiate the responses of people with different levels of family cohesion. Items 11 and 19, which presented the highest discrimination parameters, refer to the preference for sharing leisure time with the family and that the family unit is essential. This is expected, as family participation in leisure activities has been suggested to have a significant relation with family quality of life [46] and family cohesion [47]. In addition, personal beliefs about the family unit are shown to be linked to a higher level of family resilience [48]. In the adaptability factor, all its items allow us to adequately differentiate the responses of people with different levels of family adaptability. Especially items 7 and 6, which presented the highest discrimination parameters, refer to two essential aspects: (a) when faced with a problem, the family usually negotiates to find a solution, and (b) the children’s opinion is taken into account to develop family discipline guidelines. In relation to this, seeking a consensus among family members to face a problem favors family adaptation to new contexts [49]. In addition, assertive family discipline favors better coexistence and family adaptability [50]. Regarding the difficulty indexes, the items of both factors indicated increasing monotonic values, i.e., people with low levels of cohesion and adaptability choose the first or second category. As they have a higher level of the trait, they will choose higher categories. This pattern reflects the fact that the content of each of the 20 items makes it possible to take advantage of all the response alternatives and that there is no loss of information.

In relation to DIF, in the cohesion factor, items 5, 8, 13, 17 and 19 presented DIF between university students from Colombia and Chile, with a greater discriminatory power for the Chilean group. These items better distinguish low and high levels of cohesion in Chilean students. Furthermore, it indicates that Colombian students require a higher level of cohesion to answer the higher categories in items 19, 17, and 13. By contrast, Chilean students need a higher level of the latent trait to answer the higher categories in items 1, 5, and 8. Regarding the adaptability factor, only item 18 indicated a differential performance between both countries, with a greater discriminatory power for Colombian students. Considering that the social and cultural aspect is closely linked to family functioning [51], the cultural, economic, and educational differences of both countries could cause different interpretations of the family functioning items, especially in the items presenting DIF.

This study has several limitations. First, a non-probabilistic convenience sample was used, limiting the generalizability of the results to both countries. In addition, both groups had a higher predominance of women and young participants (< 30 years). Further, differences were observed in terms of sample sizes between countries, with Colombia having larger sample sizes. Therefore, future studies should use probability sampling techniques and larger and more representative samples for both countries. Third, DIF was not assessed according to sex and age due to the sample size. Therefore, future studies should perform a DIF analysis for these groups.

Despite these limitations, the items of FACES III present adequate psychometric performance both in terms of their discriminative capacity and difficulty. Therefore, the items provide helpful information on levels of cohesion and adaptability, thereby allowing a better understanding of family functioning in health science students. Notably, this is the first study to show evidence of the psychometric performance of FACES-III through the IRT in Chile and Colombia. Moreover, as indicated in the study, some items of cohesion and adaptability present differential functioning between the two countries. These results should be considered when making cross-cultural comparisons of family functioning in health sciences students in Colombia and Chile.

## Data Availability

The datasets used and/or analysed during the current study are available from the corresponding author on reasonable request.
